# Answer to Letter to the Editor Regarding the Article “Brazilian and US-American Researchers Cite Articles Written by Brazilian Authors in Revista Brasileira de Ortopedia”

**DOI:** 10.1055/s-0044-1785469

**Published:** 2024-04-10

**Authors:** Vincenzo Giordano, Juliana Lyra, João Artur Bonadiman, Osvandré Lech

**Affiliations:** 1 Serviço de Ortopedia e Traumatologia Prof. Nova Monteiro, Hospital Municipal Miguel Couto, Rio de Janeiro, RJ, Brasil; 2 Clínica São Vicente, Rede D'or São Luiz, Rio de Janeiro, RJ, Brasil; 3 Instituto de Ortopedia e Traumatologia, Hospital São Vicente de Paulo, Passo Fundo, RS, Brasil

We thank the colleagues for their contribution to the information presented in our 2021 study, corroborating a previous observation made by one of the authors of that article. 1 2 Certainly the study carried out by the colleagues presents a broader evaluation and uses statistical tests that clearly show that Brazilian and North American authors consider Brazilian orthopaedic research in a similar way. 3 This finding is of extreme relevancy, showing that colleagues from another country consider our scientific production. Undoubtedly this reflects one of the great achievements carried out in recent years by placing our largest journal on the largest scientific data search platform in the world.

In the study carried out by colleagues, an increase in the number of articles published by the Revista Brasileira de Ortopedia over the seven years included in **Table 1** is clearly observed, going from 98 in 2013 to 131 in 2020. In parallel, it is noted that the number of citations to Brazilian authors also increased, going from 17 in 2013 to 123 in 2020. Interestingly, the number of articles by Brazilian authors in this same period did not increase significantly, going from 93 in 2013 to 109 in 2020. In the same way, it is observed that the number of citations (excluding self-citations) increased from 2013 (0.097) to 2020 (0.248), however it still seems to us that it remains quite low for the quality of scientific production developed in our country. 3

It was exactly this aspect what we wanted to raise and bring to the discussion in our article, as currently the most used model both for evaluating scientific journals and the visibility of what is published by the author continues to be the impact factor (IF). IF was proposed the “journal impact factor” to evaluate journals in the Citation Index and to assist librarians in choosing which journals to subscribe to. 4 In a relatively simple formula, a journal's IF is an average of the citations that papers published in the previous two years attracted that year. Although it has become a staple in many types of analyses of a journal's scientific impact, recently the application of the journal impact factor in politics and decision-making in academia has been criticized as it can often be based on false beliefs and unjustified inferences. 5 For sure, a more effective indicator could involve considering the total number of citations a publication receives from its year of publication up to the end of the most recent year. 6

While this aspect remains an active and critical point of discussion in academia, including the fact that it is a scientometric index calculated by Clarivate and indexed by the Clarivate Web of Science, in our study we wanted to address which strategies that can be employed so that we can gradually, but solidly, increase the number of citations from Brazilian authors to Brazilian authors. The current study corroborates our thoughts that only the development and implementation of strong strategies can improve the visibility of the journal in the world academic-scientific scenario.
