# Supplementary material for: Answer to Letter to the Editor Regarding the Article “Brazilian and US-American Researchers Cite Articles Written by Brazilian Authors in Revista Brasileira de Ortopedia”
Source: Rev Bras Ortop (Sao Paulo). 2024 Apr 10;59(2):e334–5. doi: 10.1055/s-0044-1785469 (PMC11006507; doi:10.1055/s-0044-1785469)
Supplement: Related texts

# Research article: Resposta à carta ao editor referente ao artigo “Pesquisadores brasileiros e norte-americanos citam artigos escritos por autores brasileiros na Revista Brasileira de Ortopedia”

Agradecemos aos colegas pela contribuição nas informações apresentadas em nosso estudo de 2021, corroborando observação anterior feita por um dos autores daquele artigo. 1 2 Certamente o estudo realizado pelos colegas apresenta uma avaliação mais ampla e utiliza testes estatísticos que mostram claramente que autores brasileiros e norte-americanos consideram a pesquisa ortopédica brasileira de forma semelhante. 3 Esse achado é de extrema relevância, mostrando que colegas de outro país consideram nossa produção científica. Sem dúvida isso reflete uma das grandes conquistas alcançadas nos últimos anos ao colocar o nosso maior periódico na maior plataforma de busca de dados científicos do mundo.

No estudo realizado pelos colegas, observa-se claramente um aumento no número de artigos publicados pela Revista Brasileira de Ortopedia ao longo dos sete anos incluídos na **Tabela 1** passando de 98 em 2013 para 131 em 2020. Paralelamente, nota-se que o número de citações a autores brasileiros também aumentou, passando de 17 em 2013 para 123 em 2020. Curiosamente, o número de artigos de autores brasileiros neste mesmo período não aumentou significativamente, passando de 93 em 2013 para 109 em 2020. Da mesma forma, observa-se que o número de citações (excluindo autocitações) aumentou de 2013 (0,097) para 2020 (0,248), porém ainda nos parece que permanece bastante baixo para a qualidade da produção científica desenvolvida em nosso país. 3

Foi exatamente esse aspecto que queríamos levantar e trazer para a discussão em nosso artigo, pois atualmente o modelo mais utilizado tanto para avaliação de revistas científicas quanto para visibilidade do que é publicado pelo autor continua sendo o fator de impacto (FI). Foi proposto o “fator de impacto do periódico” para avaliar periódicos no Citation Index e para auxiliar os bibliotecários na escolha de quais periódicos assinar. 4 Numa fórmula relativamente simples, o FI de uma revista é uma média das citações que os artigos publicados nos dois anos anteriores atraíram naquele ano. Embora tenha se tornado um elemento básico em muitos tipos de análises do impacto científico de um periódico, recentemente a aplicação do fator de impacto do periódico na política e na tomada de decisões na academia tem sido criticada, pois muitas vezes pode ser baseada em crenças falsas e inferências injustificadas. 5 Com certeza, um indicador mais eficaz poderia envolver o número total de citações que uma publicação recebe desde o ano de publicação até o final do ano mais recente. 6

Embora esse aspecto continue sendo um ponto ativo e crítico de discussão no meio acadêmico, inclusive por se tratar de um índice cienciométrico calculado pela Clarivate e indexado pela Clarivate Web of Science, em nosso estudo quisemos abordar quais estratégias podem ser empregadas para que possamos aumentar gradativamente, mas de forma sólida, o número de citações de autores brasileiros por autores brasileiros. O presente estudo corrobora nossos pensamentos de que somente o desenvolvimento e implementação de estratégias fortes podem melhorar a visibilidade da revista no cenário acadêmico-científico mundial.
